# Malignant Phyllodes Tumour with Liposarcomatous Differentiation, Invasive Tubular Carcinoma, and Ductal and Lobular Carcinoma In Situ: Case Report and Review of the Literature

**DOI:** 10.4061/2010/501274

**Published:** 2010-07-05

**Authors:** Mardiana Abdul Aziz, Frank Sullivan, Michael J. Kerin, Grace Callagy

**Affiliations:** ^1^Division of Anatomic Pathology, Galway University Hospitals, Galway, Ireland; ^2^Department of Radiation Oncology, Galway University Hospitals, Galway, Ireland; ^3^Department of Surgery, Galway University Hospitals, Galway, Ireland; ^4^Division of Pathology, Clinical Science Institute, National University of Ireland, Galway, Costello Road, Galway, Ireland

## Abstract

A 43-year-old woman presented with a right breast lump that had enlarged over 5 months. She had chemoradiotherapy for non-Hodgkin's lymphoma in 1989. Histology revealed a malignant phyllodes tumour (PT) with liposarcomatous differentiation and ductal carcinoma in situ (DCIS) within the tumour with invasive tubular carcinoma, DCIS, and lobular carcinoma in situ in the surrounding breast. She had surgery and adjuvant radiotherapy. One year follow-up showed no recurrence or metastatic disease. Liposarcomatous differentiation is uncommon in PTs, and coexisting carcinoma is rare with 38 cases in 31 reports in the literature. Carcinoma is reported in malignant (*n* = 19), benign (*n* = 16) and in borderline PTs (*n* = 3) with invasive carcinoma (*n* = 18) and pure in situ carcinoma (*n* = 19) recorded in equal frequency. Carcinoma is more commonly found within the confines of benign PTs; whereas it is more often found surrounding the PT or in the contralateral breast in malignant PTs. Previous radiotherapy treatment is reported in only two cases. The aetiology of co-existing carcinoma is unclear but the rarity of previous radiotherapy treatment suggests that it is incidental. This case highlights the diverse pathology that can occur with PTs, which should be considered when evaluating pathology specimens as they may impact on patient management.

## 1. Introduction

Phyllodes tumors (PTs) of the breast are uncommon biphasic fibroepithelial neoplasms that account for <1% of all breast tumours. Most PTs are benign and carry a risk of local recurrence whereas malignant PTs have a 13% risk of haematogenous metastasis [1].The distinction between benign, borderline and malignant PT is based on the assessment of a number of histological features including infiltrative margin, stromal overgrowth, stromal atypia, cellularity, and mitotic activity.However,while histological features are helpful, they are not accurate predictors of tumour behavior, and no single parameter is reliable in all cases [2].

PTs are believed to arise from intralobular or periductal stroma and may arise de novo or from pre-existing fibroadenomas [2]. Up to 30% of PTs show malignant transformation, most often in the form of malignant transformation of the stroma, which usually shows fibrosarcomatous differentiation and rarely heterologous sarcomatous elements. Malignant transformation of epithelial elements is very rare with only 38 cases reported in the literature. We present a case of a malignant PT that contained heterologous liposarcomatous stromal differentiation and exhibited a range of epithelial pathology. Ductal carcinoma in situ (DCIS) was present within the PT; DCIS, invasive tubular carcinoma, and multiple foci of lobular carcinoma in situ (LCIS) were noted in the adjacent breast tissue. Although it is generally accepted that the prognosis of a patient with a malignant PT depends on the nature of the PT, malignant epithelial components should, if present, be taken into account when managing the patient.

## 2. Case Report

**Figure 1:**
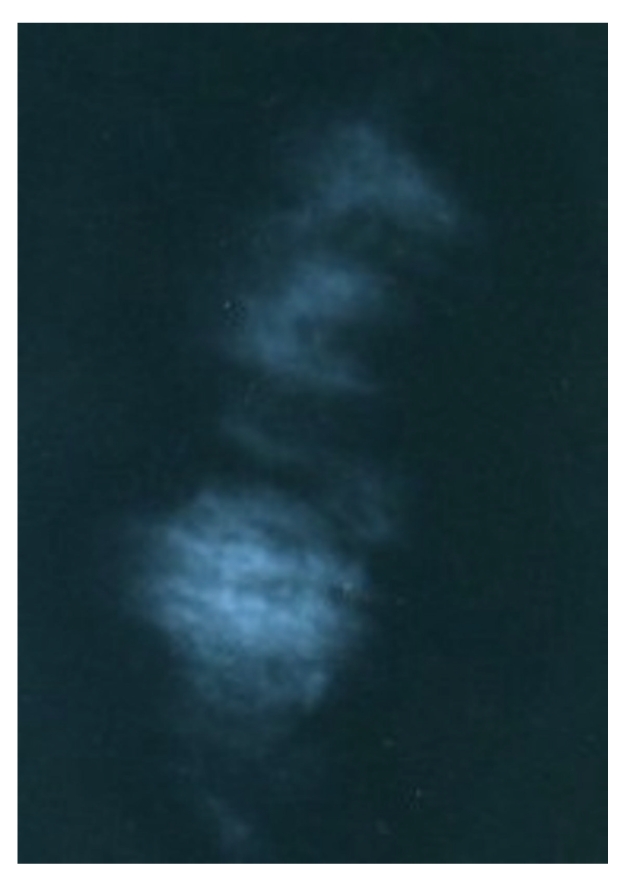
Mammogram of the left breast. A relatively well-circumscribed mass was present in lower central aspect of the breast.

A 43-year-old woman presented with a palpable lump in the central aspect of the left breast below the nipple. The lump was present for five months. It had increased in size over that time and was tender. Mammography showed a relatively well-circumscribed 3.4 cm mass in the lower central aspect of the breast (Figure 1). An ultrasound-guided biopsy was performed and showed a fibroepithelial lesion with increased stromal cellularity and nuclear atypia, consistent with a PT. The patient had a history of non-Hodgkin's lymphoma involving the lumbar spine in 1989, which was treated with chemotherapy and radiotherapy to the lumbar area.

A wide local excision of the left breast was performed which was followed by a wider cavity excision and latissimus dorsi flap reconstruction. The patient was given adjuvant radiotherapy. The breast received 50.4 Gy at 1.8 Gy per fraction with a cone-down boost to the tumour bed, giving a total dose of 63 Gy to the area considered at the greatest risk of recurrence. Follow-up after one year showed no evidence of recurrence or metastasis. Axillary sampling was not performed but no axillary lymphadenopathy was identifiedat one year follow-up.

## 3. Pathological Findings

**Figure 2:**
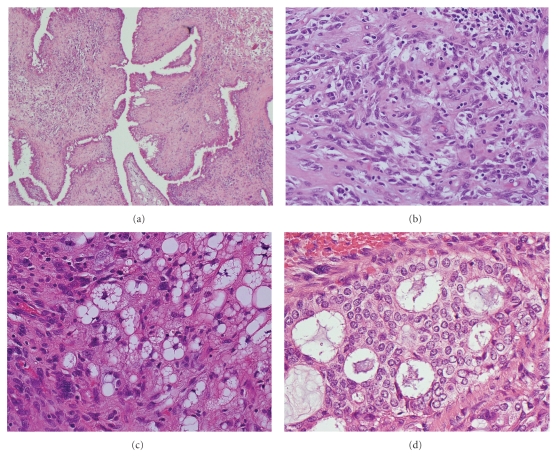
Pathological features within phyllodes tumour: (a) leaf-like architecture of the phyllodes tumour (H&E, original magnification 40x); (b) fibrosarcomatous stroma (H&E, original magnification 100x); (c) liposarcomatous differentiation in the stroma. Note the presence of lipoblasts (H&E, original magnification 200x); (d) DCIS, intermediate nuclear grade (H&E, original magnification 400x).

**Figure 3:**
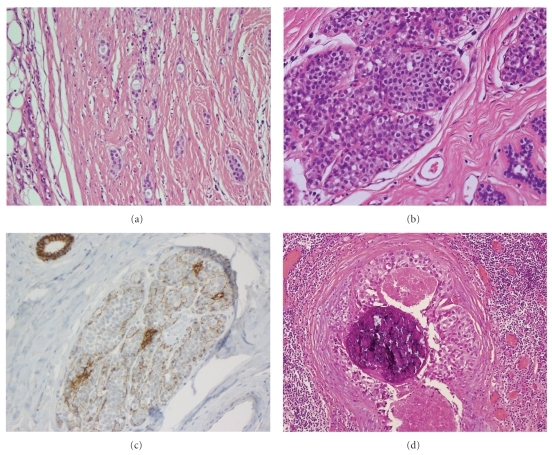
Pathological features in ipsilateral breast peripheral to phyllodes tumour(a)Invasive tubular carcinoma (H&E, original magnification 200x); (b) LCIS (H&E, original magnification 200x); (c) LCIS showing downregulation in E-Cadherin staining (E-Cadherin, original magnification 200x); (d) DCIS, high nuclear grade with comedonecrosis and calcification (H&E, original magnification 200x).

The wide local excision of the left breast, 75 × 60 × 30 mm, contained a well-defined, partly encapsulated tumor, 25 × 35 × 27 mm, with a papillary texture. A PT was confirmed histologically. The tumour showed the characteristic enhanced intracanalicular growth pattern with leaf-like projections into dilated cysts, extensive stromal overgrowth, and marked stromal hypercellularity (Figure 2(a)). Frankly malignant stromal features including nuclear pleomorphism and a high mitotic count (19 *per* 10 high power fields) were seen (Figure 2(b)). In many areas, liposarcomatous differentiation, characterized by pleomorphic lipoblasts, was present (Figure 2(c)). The margins of the PT were focally infiltrative. The epithelial component within the PT exhibited hyperplasia of usual type as well as foci of DCIS, intermediate grade, with cribriform and solid growth patterns, without necrosis (Figure 2(d)). A small invasive carcinoma, 2 mm in size, was present at the periphery of the PT close to the deep margin of the specimen (Figure 3(a)). This was composed of well-formed tubules that lacked a myoepithelial layer (Smooth Muscle Heavy Chain Myosin and p63 negative) and was regarded as a grade 1 tumour [3] although it was too small to grade accurately based on the absence of sufficient high power fields for scoring of mitotic activity. Oestrogen receptor was strongly expressed and Her-2 was negative. The invasive tumour was adjacent to a small duct with morphological features of low-grade DCIS but there was no morphological transition between invasive carcinoma and the stromal or epithelial components of the adjacent PT. Multiple scattered foci of LCIS were also present peripheral to the PT, confirmed by downregulation of E-Cadherin staining (Figures 3(b) and 3(c)). The scattered nature of the LCIS precludes accurate measurement of its size.

The cavity re-excision specimen (190 g, 98 × 97 × 47 mm) included skeletal muscle and showed no residual PT. A single focus of DCIS, high-nuclear grade with comedo-necrosis and microcalcification, 3 mm in size, was present within 1 mm to the medial margin (Figure 3(d)). Multiple foci of LCIS were also noted.

## 4. Discussion and Review

Metaplastic change within PTs is uncommon. In the largest series of PTs reported, stromal metaplasia was present in only 11 of 335 cases, and malignant transformation of epithelium in the form of DCIS and LCIS was seen in only two cases [17]. Stromal changes included adipose and chondromyxoid elements and were seen in benign and borderline PTs whereas malignant heterologous elements were reported in malignant PTs. The latter was most commonly in the form of liposarcomatous differentiation [17, 20, 24, 30, 33, 34], which does not equate with more aggressive clinical behavior [30, 35]. Other forms of heterologous change are reported in PTs including osteosarcoma, rhabdomyosarcomas, leiomyosarcoma, rhabdomyosarcoma, and angiosarcoma [17, 36]. Epithelial change in the form of usual-type epithelial hyperplasia is well recognized in PTs [17] but epithelial metaplasia is uncommon. Apocrine and squamous changes can occur but malignant epithelial transformation, as reported in the present case, is exceptionally rare. This case adds to the literature of 38 cases in 31 reports [1, 4–33] of carcinoma arising either within and/or in association with PT (Table 1). It demonstrates the range of changes that can be seen in association with a PT, including both liposarcomatous stroma and DCIS within the tumour and DCIS, LCIS, and invasive carcinoma in the peritumoural ipsilateral breast.

Malignant epithelial elements are reported in all categories of PT. In situ and invasive carcinoma may involve the tumour itself and/or coexist with a PT elsewhere in the same or contralateral breast. Carcinoma is most commonly reported (*n* = 19) in malignant PTs. In the malignant PT category, carcinoma occurred more frequently in the ipsilateral [11, 20, 25, 30, 32] or contralateral [22, 26, 29–31] breast rather than within the confines of the PT (*n* = 7) [17, 21, 23, 24, 27, 28, 33]. Coexisting carcinoma in malignant PTs is more commonly ductal (invasive ductal carcinoma, *n* = 6; DCIS, *n* = 10) than lobular phenotype (invasive lobular carcinoma, *n* = 2; LCIS, *n* = 4) [11, 17, 19–33]. Heterologous stroma, most often liposarcomatous differentiation, was common in malignant PTs where carcinoma was also found (*n* = 8, 47%) [20, 21, 24, 27, 30, 33]. From the available data, five patients with malignant PTs with coexisting carcinoma died from metastatic PT, between three months to 51 months after diagnosis [11, 20, 21, 25, 27], and one patient had metastatic carcinoma in two lymph nodes [19].

Carcinoma is less common in benign PTs compared with malignant PTs, given the higher prevalence of the former. Coexistent carcinoma is reported in 16 benign PTs (including four of unspecified malignant potential) [7, 13, 17] and in three of these cases it was only seen in a recurrence of a benign PT [11, 13, 14]. Carcinoma was found more commonly within the benign PT (*n* = 10) [1, 4–6, 8–10, 12, 14, 17] rather than peripheral to it in the ipsilateral (*n* = 5) [7, 11, 15–17] or contralateral breast (*n* = 1) [13]. The majority of these carcinomas (*n* = 10) were invasive, with or without an in situ component and most were ductal NST type (*n* = 6) [1, 7, 8, 11, 13, 15] with individual reports of invasive lobular [6], papillotubular [12], and tubular carcinoma [14]. One case had both invasive squamous carcinoma and invasive ductal carcinoma showing clear cell, secretory, and squamous differentiation [5]. Two patients died from breast disease [11, 15], one of which had metastatic carcinoma in lymph nodes [15]. Another patient had metastatic carcinoma in four lymph nodes but remained alive and well at follow-up [1].

There are only three reports of carcinoma (DCIS) arising in association with borderline PT. Two patients had DCIS both within the PT and in the ipsilateral breast [11, 18], one of whom died within three years from unrelated causes. In the other case, DCIS occurred in the ipsilateral breast at the time of diagnosis of a recurrent PT and death occurred from metastatic PT one year after the recurrence [18].

The molecular events involved in transformation and progression of PTs are largely unknown, and the rarity of coexisting epithelial malignancy makes it difficult to draw conclusions about the aetiological association between the carcinoma and PT in these cases. Genetic aberrations have been consistently demonstrated in PTs with increasing frequency from benign to borderline to malignant PTs. Most studies to date focused on stromal alterations and showed recurrent copy number gains and losses at +1q, −13q, −6q, +5, and −10p [37–39]. Where epithelium was evaluated separately, distinct molecular alterations were demonstrated in the Wnt2-APC-B-catenin pathway, and a role was postulated for both stroma and epithelium in the neoplastic process [40, 41]. It is unclear, however, if the malignant transformation of epithelium results from stromal-epithelial interactions within the PT or if it represents cancerisation of a PT by carcinoma arising in the duct system peripheral to the PT. The latter may play a role in cases where carcinoma is present within the PT as well as peripheral to it in the ipsilateral breast. The finding of carcinoma in benign, borderline, and malignant PT and within fibroadenomas suggests that it is unlikely to be directly related to the number of genetic aberrations in the different categories of PT. A role of exogenous carcinogen exposure in malignant transformation merits consideration as an aetiological agent because two patients [10, 20] in addition to the present case had a history of previous radiation. In all cases the radiotherapy field was remote from the breast which suggest that it is likely to be incidental.

The present case taken together with those in the literature demonstrates the diverse pathology that can be found with PTs, both within the tumour and in the surrounding and/or contralateral breast. The distinction between a malignant PT with coexisting carcinoma and a metaplastic carcinoma or carcinosarcoma should be considered in the diagnosis as these entities are managed differently and the distinction affects patient outcome. Carcinosarcomas have mixed malignant epithelial and stromal components with the latter showing no reactivity for epithelial immunohistochemical markers. While the present case fulfils these criteria, the characteristic leaf-like structure with malignant heterologous differentiation favours a diagnosis of malignant PT with coexisting carcinoma rather than a true carcinosarcoma. Carcinomas coexisting with PTs are generally diagnosed incidentally on the wide local excision, and the prognosis is dictated by the category of PT. In the present case, the malignant PT and not the carcinomatous elements will have the greatest impact on prognosis. However, the presence of coexistent carcinoma must be taken into account in management decisions, especially for benign and borderline PTs. Wider adequacy of excision of carcinoma, as in this case, and lymph node sampling should be considered.

## Figures and Tables

**Table 1:** Summary of cases of phyllodes tumours associated with carcinoma.

Report	Age	PT type	Size (mm)	Associated carcinoma		Location of carcinoma relative to PT	Comment	Outcome
				Invasive	In situ			
Yamaguchi et al. [4]	54	BPT	150		DCIS	Within		AW at 11 months
Ramdass and Dindyal [5]	69	BPT	NS	IDC		Within	Complex carcinoma with clear cell, secretory and squamous differentiation	NA

Parfitt et al. [1]	26	BPT	33	IDC	DCIS	Within	Metastatic adenocarcinoma in 4 of 13 LN	AW at 36 months

Kodama et al. [6]	47	BPT	170	ILC	DCIS	Within	Lump present for 12 years before treatment	AW at 108 months

De Rosa et al. [7]	77	BPT*	50	IDC	DCIS	Ipsilateral	Mild atypia in stroma with occasional mitoses	AW at 10 months
Yasumura et al. [8]	47	BPT	130	IDC		Within		AW at 66 months
Knudsen and Ostergaard [9]	71	BPT	70		DCIS, LCIS	Within		NA
Grove and Deibjerg Kristensen [10]	71	BPT	190		DCIS	Within	History of irradiation to ovaries for climacteric menstrual disorders	AW at 4 months

Christensen et al. [11]	42–58	BPT	10–20	IDC		Ipsilateral	IDC in close relation to a recurrent PT	RIP after 3 months from metastatic carcinoma
Ishida et al. [12]		BPT	56	Papillotubular		Within		AW at 30 months

Stone-Tolin et al. [13]	59	BPT/NS	220	IDC		Contralateral	Recurring PT and FA over 36-year period	AW at 15 months

Leong and Meredith [14]	47	BPT	40	ITC		Within	Three recurrences of BPT. LCIS in second recurrence and ITC in third recurrence	AW at 21 months

Richards and WAY [15]	37	BPT	70	IDC**		Ipsilateral	Separate tumour nodules. Metastatic carcinoma in LN	RIP after 9 months from metastases^†^

Lester and Stout, [16]	40	BPT	14	NS	NS	Ipsilateral	PT found in mastectomy specimen performed for carcinoma	AW at 144 months
Tan et al. [17]	NA	NS^∞^	NA		DCIS	Ipsilateral		NA
Tan et al. [17]	NA	NS^∞^	NA		LCIS	Within		NA
Deodhar et al. [18]	51	BLPT	140		DCIS	Within, Ipsilateral		NA
Christensen et al. [11]	42–58	BLPT	NS		DCIS	Within, Ipsilateral		RIP after 36 months from unrelated cause
Christensen et al. [11]	42–58	BLPT	NS		DCIS	Ipsilateral	Recurrent PT associated with DCIS in adjacent breast within 12 months of initial diagnosis	RIP after 12 months from metastatic PT

Korula et al. [19]	51	MPT	210		DCIS	Within, Ipsilateral	Metastatic carcinoma in 2 of 12 lymph nodes	AW at 11 months
Kefeli et al. [20]	26	MPT	45	IDC		Ipsilateral	Liposarcomatous and chondrosarcomatous stroma in PT; history of osteosarcoma and radiotherapy	RIP after 12 months^‡^

Sugie et al. [21]	54	MPT	60	IDC	DCIS	Within	Carcinoma showed squamous differentiation	RIP after 40 months from metastatic PT

Merck et al. [22]	NS	MPT	NS	IDC		Contralateral		AW at 32 months
Nomura et al. [23]	75	MPT	35		DCIS	Within		AW at 32 months
Lim and Tan [24]	45	MPT	120		DCIS	Within	Liposarcomatous differentiation in PT; two FAs in contralateral breast	RIP after 108 months from unrelated cause

Tan et al. [17]	NS	MPT	NS		DCIS	Within		
Auerbach [25]	69	MPT	NS	IDC		Ipsilateral	PT recurred after 40 months with metastases	RIP after 51 months from metastases^†^

Gebrim et al. [26]	58	MPT	300	ILC		Contralateral	ILC within FA in contralateral breast	AW at 84 months

Nishimura et al. [27]	80	MPT	105		DCIS	Within	Osteosarcomatous, rhabdomyosarcomatous, fibrosarcomatous stroma in PT	RIP after 3 months from metastases^†^

Padmanabhan et al. [28]	47	MPT	75		LCIS	Within	Liposarcomatous and fibrosarcomatous stroma in PT	AW at 6 months

Kasami et al. [29]	47	MPT	NS	ILC		Contralateral	46XX/46XY mosaic karyotype; three sisters with breast carcinoma. Recurrent PT at autopsy	NA

Powell and Rosen [30]	17–71	MPT	8–100		DCIS	Ipsilateral	Liposarcomatous stroma in PT	NA

Powell and Rosen [30]	17–71	MPT	8–100		LCIS	Contralateral	Liposarcomatous stroma in PT	NA

Powell and Rosen [30]	17–71	MPT	8–100	IDC	DCIS	Ipsilateral, Contralateral	Initially BPT, recurred as MPT with liposarcomatous differentiation in PT; Ipsilateral DCIS; Contralateral IDC	NA

Morimoto et al. [31]	49	MPT	110		LCIS	Contralateral	LCIS within contralateral FA	AW at 132 months
Christensen et al. [11]	42–58	MPT			LCIS	Ipsilateral		RIP after 12 months from metastatic PT
Huntrakoon [32]	31	MPT	90	IDC	DCIS	Ipsilateral		AW at 24 months
Seemayer et al. [33]	27	Stromal sarcoma***	60		DCIS	Within	Liposarcomatous differentiation in PT. Contralateral MPT after initial mastectomy	NA

Current case	43	MPT	35	IDC	DCIS, LCIS	Within, Ipsilateral	Liposarcomatous differentiation in PT. DCIS within PT. IDC and LCIS in ipsilateral breast. Previous radiotherapy for lymphoma.	AW at 12 months

*Category of PT not specified in original report, interpreted as benign PT.

**Classified as scirrhous adenocarcinoma.

***Sarcomatous stroma with liposarcomatous differentiation, tumour lacked circumscription and features of PT, for example, leaf-like structures were not present.

^†^Metastatic component (sarcoma versus carcinoma) not specified.

^‡^Cause not specified.

^∞^Category of PT not specified.

AW: alive and well: BPT, benign PT: BLPT, borderline PT: DCIS, ductal carcinoma in situ: FA, fibroadenoma: IDC, invasive ductal carcinoma: ILC, invasive lobular carcinoma: ITC, invasive tubular

carcinoma: LCIS, lobular carcinoma in situ: LN, lymph node: MPT, malignant PT: NA, not available: NS, not specified.
